# CANPT Score: A Tool to Predict Severe COVID-19 on Admission

**DOI:** 10.3389/fmed.2021.608107

**Published:** 2021-02-18

**Authors:** Yuanyuan Chen, Xiaolin Zhou, Huadong Yan, Huihong Huang, Shengjun Li, Zicheng Jiang, Jun Zhao, Zhongji Meng

**Affiliations:** ^1^Department of Infectious Diseases, Taihe Hospital, Hubei University of Medicine, Shiyan, China; ^2^Institute of Biomedical Research, Taihe Hospital, Hubei University of Medicine, Shiyan, China; ^3^Department of Liver Diseases, Yichang Central People's Hospital, China Three Gorges University, Yichang, China; ^4^Department of Liver Diseases, HwaMei Hospital, University of Chinese Academy of Sciences, Ningbo, China; ^5^Department of Infectious Diseases, Ankang Central Hospital, Hubei University of Medicine, Ankang, China; ^6^School of Public Health, Hubei University of Medicine, Shiyan, China; ^7^Hubei Clinical Research Center for Precise Diagnosis and Treatment of Liver Cancer, Shiyan, China

**Keywords:** SARS-CoV-2, COVID-19, severe illness, prediction, nomogram

## Abstract

**Background and Aims:** Patients with critical coronavirus disease 2019 (COVID-19) have a mortality rate higher than 50%. The purpose of this study was to establish a model for the prediction of the risk of severe disease and/or death in patients with COVID-19 on admission.

**Materials and Methods:** Patients diagnosed with COVID-19 in four hospitals in China from January 22, 2020 to April 15, 2020 were retrospectively enrolled. The demographic, laboratory, and clinical data of the patients with COVID-19 were collected. The independent risk factors related to the severity of and death due to COVID-19 were identified with a multivariate logistic regression; a nomogram and prediction model were established. The area under the receiver operating characteristic curve (AUROC) and predictive accuracy were used to evaluate the model's effectiveness.

**Results:** In total, 582 patients with COVID-19, including 116 patients with severe disease, were enrolled. Their comorbidities, body temperature, neutrophil-to-lymphocyte ratio (NLR), platelet (PLT) count, and levels of total bilirubin (Tbil), creatinine (Cr), creatine kinase (CK), and albumin (Alb) were independent risk factors for severe disease. A nomogram was generated based on these eight variables with a predictive accuracy of 85.9% and an AUROC of 0.858 (95% CI, 0.823–0.893). Based on the nomogram, the CANPT score was established with cut-off values of 12 and 16. The percentages of patients with severe disease in the groups with CANPT scores <12, ≥12, and <16, and ≥16 were 4.15, 27.43, and 69.64%, respectively. Seventeen patients died. NLR, Cr, CK, and Alb were independent risk factors for mortality, and the CAN score was established to predict mortality. With a cut-off value of 15, the predictive accuracy was 97.4%, and the AUROC was 0.903 (95% CI 0.832, 0.974).

**Conclusions:** The CANPT and CAN scores can predict the risk of severe disease and mortality in COVID-19 patients on admission.

## Introduction

Coronavirus disease 2019 (COVID-19) is caused by severe respiratory syndrome coronavirus 2 (SARS-CoV-2), and pneumonia is the main clinical manifestation (1–3). SARS-CoV-2 is highly transmissible (4, 5), and the COVID-19 pandemic has spread to every country. With the rapid increase in the number of confirmed cases, medical resources have been inadequate (6).

As of December 30, 2020, the cumulative number of confirmed cases worldwide exceeded 80 million, and more than 1.78 million patients had died; the daily number of newly diagnosed patients is still rising. Although many clinical trials have been performed in the treatment of COVID-19 patients, so far, only dexamethasone has been validated in reducing the mortality rate of critically ill patients, and no specific medicine is available for COVID-19 (7). The demand for intensive care unit (ICU) beds, ventilators, protective equipment, other medical resources and medical staff exceed the existing supply by 10-fold (6). The early identification of patients at risk for severe disease and death, the timely initiation of interventions and admission to the ICU can prevent disease progression and reduce the mortality rate. Patients with mild COVID-19 require access to only limited medical resources for isolation and general symptomatic treatment. Therefore, it is very important to establish models predicting the prognoses of patients with COVID-19. More than 700 prognosis-related articles have been published in journals and on preprint platforms; most articles have only provided the risk factors for a poor outcome in COVID-19 patients; and ~50 prognostic models have been reported (8). Yuan et al. reported a model with good predictive efficacy [area under the receiver operating characteristic curve (AUROC) = 0.901] in predicting the risk of mortality in patients with COVID-19 using chest computed tomography (CT) scores. However, this model is not sufficiently representative and generalizable because only 27 patients from Wuhan were included (9). Other models were constructed using data only from patients in Wuhan (9–13), and many models have been presented as nomograms, which are inconvenient for clinical application and have not been verified in populations of other COVID-19 patients (12, 14–16).

Liang et al. established a model based on 1,590 COVID-19 patients from 31 provinces in China and validated this model in another 710 patients with COVID-19; the model is available on a web page (http://118.126.104.170/). The model showed good predictive ability for a poor prognosis of COVID-19 in both the development cohort and the external validation cohort (AUROC = 0.880). The following 10 variables were included the model: X-ray abnormalities, age, hemoptysis, dyspnea, unconsciousness, number of comorbidities, cancer history, neutrophil-to-lymphocyte ratio (NLR), lactate dehydrogenase (LDH) level, and direct bilirubin level; however, only the NLR, LDH level, and direct bilirubin level are biochemical indicators, which are inadequate for enabling a comprehensive evaluation of renal, heart, and coagulation function in patients with COVID-19. This model and several other models used ICU admission, invasive ventilation, and death as the composite outcome (14, 16–18), although not all patients with critical disease require ICU admission and/or invasive ventilation, and more than 50% of patients with critical disease will die (6); thus, individual models are needed for the precise prediction of severe disease or death. Wu et al. reported a model with good predictive efficacy (16) that was established based on data from 299 patients from Wuhan, China, and verified in 426 patients with COVID-19 from China, Italy, and Belgium. However, Collins et al. believed that the sample size of patients in their study was relatively small and that it was unreasonable to use 239 patients for model development and 60 patients for internal validation. The patients with a predicted risk of a poor prognosis from 21.00 to 80.00% were classified into the medium-risk group, further casting doubt regarding the basis of risk stratification in the study (19). The CALL score developed by Ji et al. includes comorbidities, lymphocytes, age, and LDH, has good predictive efficacy and is convenient for clinical use. However, the sample size in their study was relatively small, and there was no external validation (20). The NLR has been reported to be a prognostic factor in COVID-19 (21, 22); however, the NLR can only reflect the status of the immune system and is insufficient for assessing the comprehensive situation in COVID-19 patients because COVID-19 is a systemic disease (23, 24). In summary, these models have a risk of bias, and their reliability in clinical application has not been verified (8, 25). As the COVID-19 epidemic continues to spread, it is necessary to develop a reliable and clinically applicable prognostic model for COVID-19.

In this study, by comparing the demographic, clinical, and blood biochemical characteristics of COVID-19 patients with and without severe disease on admission, the risk factors for severe disease and mortality were identified, and risk prediction models for severe disease and death in COVID-19 patients were established.

## Materials and Methods

### Patient Selection

This study retrospectively included patients with COVID-19 diagnosed at Shiyan Taihe Hospital, Ankang Central Hospital, Ningbo Hwamei Hospital, and Yichang Central People's Hospital from January 22, 2020 to April 15, 2020. The criteria used for the diagnosis and classification of confirmed cases of COVID-19 were provided in the “Guidance for 2019 coronavirus disease prevention, control, diagnosis and management” (26). The clinical classifications were as follow. (1) Mild: the clinical symptoms were mild, and no pneumonia manifestations were observed on imaging. (2) Moderate: patients had symptoms, such as fever and respiratory tract symptoms, and pneumonia manifestations were observed on imaging. (3) Severe: any of the following criteria were met: (1) respiratory distress, respiration rate (RR) ≥30 breaths/min; (2) pulse oxygen saturation (SpO2) ≤ 93% on room air at rest; or (3) arterial partial pressure of oxygen (PaO2)/oxygen concentration (FiO2) ≤ 300 mmHg. In regions with a high altitude (more than 1 kilometer above sea level), the PaO2/FiO2 values were adjusted based on the following: equation of PaO2/FiO2 × [atmospheric pressure (mm Hg)/760]. Patients with >50% lesion progression within 24 to 48 h on pulmonary imaging were treated as having severe cases. And (4) Critical: any of the following criteria were met: (1) respiratory failure needing mechanical ventilation; (2) shock; or (3) other organ failure requiring monitoring and treatment in the ICU. In this study, the severe and critical cases were classified as having severe disease, while the mild and moderate cases were classified as having non-severe disease. Patients diagnosed with severe disease on admission were only included in the mortality risk analysis. This study was approved by the Medical Ethics Committee of Shiyan Taihe Hospital. The approval number is 2020KS018.

### Data Collection

Clinical data pertaining to COVID-19 patients on admission were retrieved from the medical record databases of Shiyan Taihe Hospital, Ankang Central Hospital, Ningbo Hwamei Hospital, and Yichang Central People's Hospital. The data included the patients' epidemiological histories, comorbidities, vital signs (heart rate, RR, blood pressure, and body temperature), signs and symptoms (fever), laboratory tests (liver and kidney function, routine blood tests, C-reactive protein (CRP) levels, and chest CT findings), and outcome at discharge. The patients with COVID-19 who progressed to severe or critical disease during hospitalization were included in the analysis of severe disease. Survival at discharge was the final outcome of this study. The included comorbidities were hypertension, diabetes, cardiocerebrovascular disease, malignant tumor, chronic liver disease, chronic kidney disease, and chronic lung disease.

### Statistical Analysis

The normally and non-normally distributed continuous variables are presented as the means ± standard deviations (SDs) and medians (interquartile ranges, IQRs), respectively. The categorical variables are presented as *n* (%). *t*-tests, chi-square tests and Mann-Whitney *U*-tests were used to compare the differences in various indicators between the two groups. To ensure that the variables conformed to a normal distribution to the greatest extent possible, natural logarithmic transformation was applied to the white blood cell (WBC) count, procalcitonin (PCT) level, CRP level and other variables. Then, we obtained the natural logarithm (ln) of WBC [ln (WBC)], ln (NLR), ln (PLT), ln (Alb), ln (Tbil), ln (Cr), etc. During the modeling process, variables with more than 10% missing values were excluded from the analysis, and variables with <10% missing values were addressed with multiple imputation. The independent prognostic risk factors were selected by a logistic regression analysis and included in the nomogram, which was used to establish the prediction model. For convenience in clinical application, the independent risk factors identified by the logistic regression analysis were converted into dichotomous variables with a cut-off value determined by receiver operating characteristic (ROC) curve analysis. A logistic regression was performed to determine the weight of the influence of the variables on disease progression and establish a new scoring model. The best cut-off value was determined according to the Youden index, sensitivity, specificity, predictive value, and likelihood ratio. The leave-one-out cross validation method was used for internal validation, and 1,000 bootstrap resamplings were performed. The AUROC and Hosmer-Lemeshow test were used to evaluate the predictive efficacy of the model. SPSS software, version 22.0 (SPSS, Inc., Chicago, IL, USA), was used for the data analysis. The nomogram was established using R software, version 3.6.1 (R Foundation for Statistical Computing, Vienna, Austria). A two-tailed *P* < 0.05 was considered statistically significant.

## Results

### Baseline Characteristics of COVID-19 Patients

**Figure 1:**
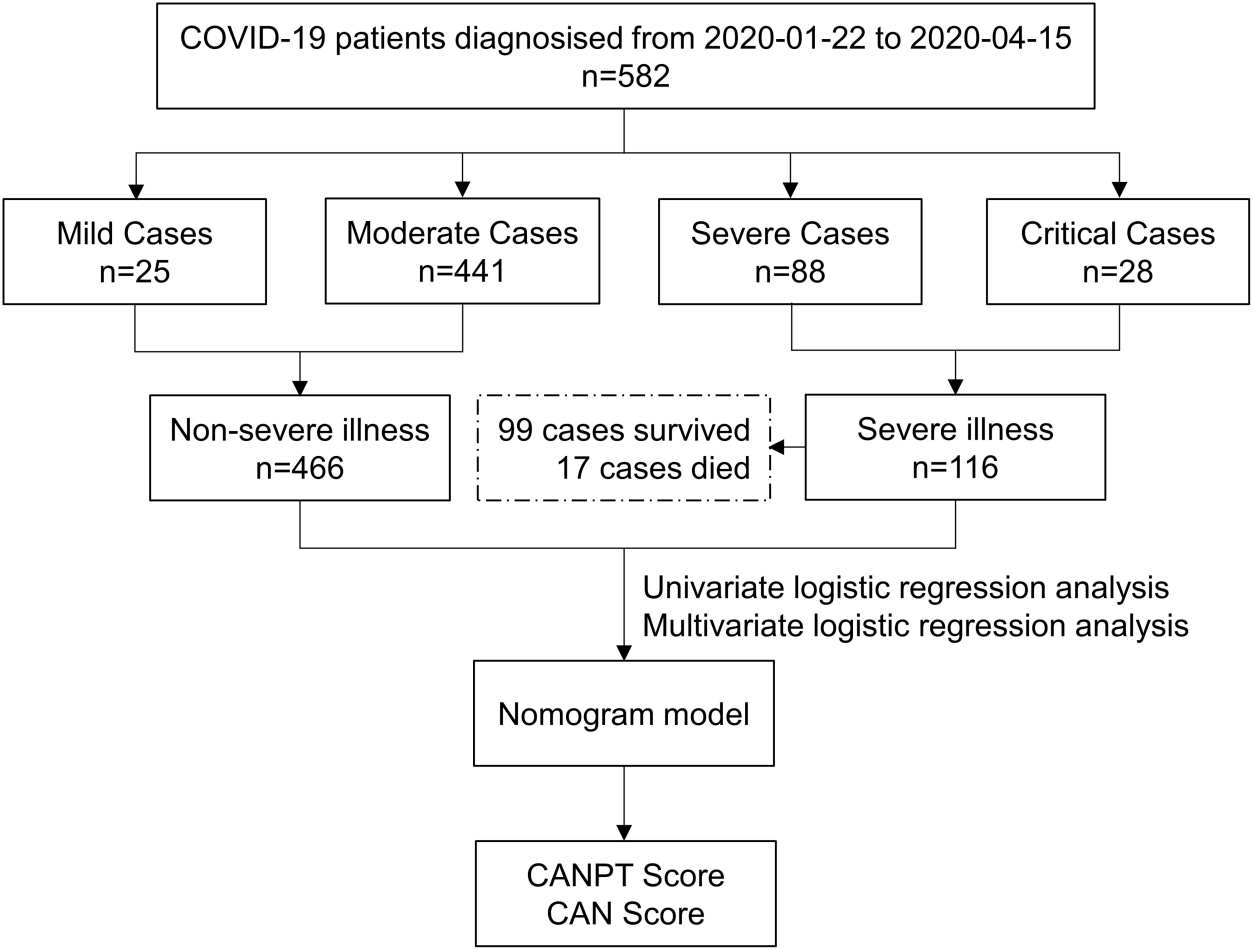
Flow chart of the study.

In total, 582 patients with COVID-19, including 202 from Shiyan Taihe Hospital, 40 from Ankang Central Hospital, 108 from Ningbo Hwamei Hospital, and 232 from Yichang Central People's Hospital, were enrolled in this study. During hospitalization, 116 patients developed severe disease, and 17 patients died. There were 466 patients with non-severe COVID-19, including 25 with mild cases and 441 with moderate cases (Figure 1). The median age of the patients with severe disease was significantly higher than that of the patients with non-severe disease (63.00 *vs*. 47.00, *P* < 0.001). The proportion of patients with comorbidities in the severe disease group was almost two times greater than that in the non-severe disease group (59.48 *vs*. 24.68%, *P* < 0.001). Hypertension was the most common comorbidity in the patients with COVID-19 (20.10%), followed by diabetes (9.28%) and malignant tumors (2.23%), which were more common in the patients with severe disease than those with non-severe disease (Table 1). Among the 582 patients with COVID-19, fever was the most common symptom (74.05%), and the incidence of fever in the patients with severe disease was higher than that in the patients with non-severe disease, although the difference was not statistically significant (80.17 *vs*. 72.53%, *P* = 0.093). However, the proportion of patients with a body temperature ≥38.5°C in the group with severe disease was significantly higher than that in the group with non-severe disease (41.38 *vs*. 22.32%, *P* < 0.001); intrapulmonary ground-glass opacities (GGOs) were observed on CT in 559 /582 (96.05%) patients with COVID-19, including all patients with severe disease, and 443/466 (95.06%) patients with non-severe disease. There was no significant difference between the two groups in terms of sex, respiration, heart rate, or blood pressure on admission (Table 1).

**Table 1:** Baseline demographics and characteristics of the COVID-19 patients.

**Characteristics**	**All patients**	**Severe disease**	**Non-severe disease**	***t/z/χ^2^* value**	***P*-value**
	***n* = 582**	***n* = 116**	***n* = 466**		
Sex (male)	286 (49.1%)	62 (53.5%)	224 (48.1%)	1.08	0.300
Age, years	50.0 (36.0, 63.0)	63.0 (50.0, 71.0)	47.0 (34.0, 57.0)	−7.72	<0.001
Heart rate, beats per minute	86.0 (78.0, 97.0)	88.0 (80.0, 101.0)	85.0 (78.0, 96.0)	−1.55	0.121
Respiratory rate, breaths per minute	20.0 (18.0, 20.0)	20.0 (18.0, 21.0)	20.0 (18.0, 20.0)	−2.16	0.033
MAP, mm Hg	93.0 (87.0, 102.0)	93.0 (87.0, 102.0)	93.0 (87.0, 102.0)	0.84	0.402
Comorbidities	184 (31.6%)	69 (59.5%)	115 (24.7%)	52.04	<0.001
Hypertension	117 (20.1%)	43 (37.1%)	74 (15.9%)	25.96	<0.001
Diabetes	54 (9.3%)	24 (20.7%)	30 (6.4%)	22.41	<0.001
Malignant tumor	13 (2.2%)	7 (6.0%)	6 (1.3%)	9.58	0.002
**Clinical symptoms**					
Fever	431 (74.1%)	93 (80.2%)	338 (72.5%)	2.82	0.093
Highest temperature ≥38.5°C	152 (26.1%)	48 (41.4%)	104 (22.3%)	17.49	<0.001

*MAP, mean arterial pressure*.

The lymphocyte (LY) count was reduced in 300/582 (51.55%) patients with COVID-19, and a reduced LY count was more common in the group with severe disease than in the group with non-severe disease (76.72 *vs*. 45.92%, *P* < 0.001). However, the group with severe disease had a significantly larger proportion of patients with elevated WBC counts, especially neutrophils (NE), than the group with non-severe disease (11.21 *vs*. 3.22%, *P* < 0.001; 23.28 *vs*. 7.73%, *P* < 0.001). Therefore, the patients with severe disease had a significantly higher NLR than those with non-severe disease (4.20 *vs*. 2.64, *P* < 0.001). The proportions of patients with reduced hemoglobin (HGB) and PLT levels were two times higher in the group with severe disease than in the group with non-severe disease (14.66 *vs*. 7.30%, *P* = 0.012; 29.31 *vs*. 13.52%, *P* < 0.001). The rates of abnormal aspartate aminotransferase (AST) and γ-glutamyl transpeptidase (GGT) levels were significantly higher in the patients with severe disease than in the patients with non-severe disease. A reduced albumin (Alb) level was more common in the patients with severe disease than those with non-severe disease (71.55 *vs*. 37.55%, *P* < 0.001). There were larger proportions of patients with abnormal levels of blood urea nitrogen (BUN), creatinine (Cr), creatine kinase (CK), LDH, D-dimer, PCT, and CRP; activated partial thromboplastin time (APTT); and erythrocyte sedimentation rate (ESR) in the group with severe disease than in the group with non-severe disease (Table 2).

**Table 2:** Baseline blood and biochemical indices of the COVID-19 patients at baseline.

**Biochemical indexes**	**Abnormal standard**	**All patients**	**Severe disease**	**Non-severe disease**	***t/z/χ^2^* value**	***P-*value**
		***n* = 582**	***n* = 116**	***n* = 466**		
**Routine blood tests**						
White blood cell count, × 10^9^/L	≥9.5	28 (4.81%)	13 (11.21%)	15 (3.22%)	12.941	<0.001
Neutrophil count, × 10^9^/L	≥6.3	63 (10.82%)	27 (23.28%)	36 (7.73%)	23.268	<0.001
Lymphocyte count, × 10^9^/L	≤ 1.1	300 (51.55%)	89 (76.72%)	214 (45.92%)	35.307	<0.001
NLR		2.86 (2.00, 4.58)	4.20 (2.50, 8.32)	2.64 (1.86, 4.18)	−6.148	<0.001
Hemoglobin, g/L	≤ 110	51 (8.76%)	17 (14.66%)	34 (7.30%)	6.291	0.012
Platelet count, × 10^9^/L	≤ 125	97 (16.67%)	34 (29.31%)	63 (13.52%)	16.675	<0.001
**Liver function**						
Alanine aminotransferase, U/L	≥40	101 (17.35%)	24 (20.69%)	77 (16.52%)	1.124	0.289
Aspartate aminotransferase, U/L	≥40	93 (15.98%)	33 (28.45%)	60 (12.88%)	16.777	<0.001
γ-glutamyl transpeptidase, U/L	≥50	89 (16.79%)	24 (26.97%)	65 (14.74%)	7.924	0.005
Alkaline phosphatase, U/L	≥100	101 (20.74%)	23 (28.05%)	78 (19.26%)	3.205	0.073
Albumin, g/L	≤ 40	258 (44.33%)	83 (71.55%)	175 (37.55%)	43.502	<0.001
Total bilirubin, μmol/L	≥21	62 (10.67%)	16 (13.91%)	46 (9.87%)	1.581	0.209
Cut-off	≥11	346 (59.45%)	79 (68.10%)	267 (57.30%)	4.500	0.034
**Renal function**						
Blood urea nitrogen, mmol/L	≥7.6	36 (6.02%)	23 (20.00%)	16 (3.43%)	40.426	<0.001
Creatinine, μmol/L	≥104	53 (9.11%)	26 (22.41%)	27 (5.79%)	30.995	<0.001
Cut-off	≥85	153 (26.29%)	51 (43.97%)	102 (21.89%)	23.362	<0.001
**Myocardium**						
Creatine kinase, U/L	≥171	82 (14.14%)	30 (25.86%)	52 (11.21%)	16.419	<0.001
Cut-off	≥104	194 (33.33%)	56 (48.28%)	138 (29.61%)	14.556	<0.001
Creatine kinase-MB, U/L	≥25	13 (2.74%)	4 (3.96%)	9 (2.41%)	0.714	0.398
Lactate dehydrogenase, U/L	≥243	165 (34.96%)	56 (55.45%)	109 (29.38%)	23.722	<0.001
**Coagulation function**						
Prothrombin time, s	≥13	65 (16.09%)	19 (20.88%)	46 (14.70%)	1.996	0.158
Activated partial thromboplastin time, s	≥36.5	68 (16.83%)	24 (26.37%)	44 (14.06%)	7.64	0.006
International normalized ratio	≥1.5	1 (0.20%)	1 (0.94%)	0 (0.00%)	3.157	0.076
D-dimer, mg/L	≥0.25	295 (66.74%)	77 (89.53%)	218 (61.24%)	24.99	<0.001
**Inflammatory indexes**						
Procalcitonin, ug/L	≥0.5	5 (1.08%)	4 (4.30%)	1 (0.27%)	8.425	<0.001
C-reactive protein, mg/L	≥5	419 (72.62%)	105 (92.11%)	314 (67.82%)	27.135	<0.001
Erythrocyte sedimentation rate, mm/1 h	≥15	244 (56.74%)	54 (66.67%)	190 (54.44%)	4.003	0.045

*Missing data of baseline blood and biochemical indices: variables with missing values exceeding 10% include: procalcitonin (118 cases), erythrocyte sedimentation rate (152 cases), alkaline phosphatase (95 cases), creatine kinase-MB (108 cases), lactate dehydrogenase (110 cases), prothrombin time and activated partial thromboplastin time (178 cases), and D-dimer (140 cases)*.

*NLR, neutrophil-to-lymphocyte ratio*.

### Independent Risk Factors for Severe COVID-19

According to univariate logistic regression analysis, 24 variables, including age, comorbidities, fever, and ln (CRP), were associated with the severity of COVID-19 and included in the multivariate logistic regression analysis (forward likelihood method). A body temperature ≥38.5°C, ln (NLR), ln (PLT), ln (Alb), ln (Tbil), ln (Cr), and ln (CK) were independent risk factors for severe COVID-19. The risk of severe illness in the patients with a body temperature ≥38.5°C was 2.37 (95% CI, 1.39, 4.03) times that in the patients with a body temperature <38.5°C. Comorbidities and high values of ln (NLR), ln (Tbil), ln (Cr), and ln (CK), and low values of ln (PLT) and ln (Alb) were associated with an increased risk of severe COVID-19 (Table 3).

**Table 3:** Risk factors for severe COVID-19 and mortality.

**Variable**	**Crude OR** **(95% CI)**	**Adjusted OR** **(95% CI)**	***P*-value**	***P*-value**
			**(Wald's test)**	**(LR-test)**
**Risk factors for severe COVID-19**				
Comorbidity	4.48 (2.93, 6.86)	2.69 (1.61, 4.49)	<0.001	<0.001
Temperature ≥38.5°C	2.46 (1.6, 3.77)	2.37 (1.39, 4.03)	0.002	0.002
ln (NLR)	2.75 (2.04, 3.71)	1.64 (1.13, 2.37)	0.009	0.008
ln (PLT)	0.27 (0.15, 0.48)	0.45 (0.23, 0.9)	0.024	0.023
ln (Alb)	0.00 (0.00, 0.00)	0.00 (0.00, 0.02)	<0.001	<0.001
ln (Tbil)	2.04 (1.29, 3.21)	1.92 (1.04, 3.52)	0.036	0.032
ln (Cr)	3.54 (1.96, 6.4)	2.68 (1.39, 5.17)	0.003	0.001
ln (CK)	1.86 (1.4, 2.48)	1.44 (1.03, 2.02)	0.034	0.034
**Risk factors for mortality**				
ln (NLR)	3.78 (2.17, 6.60)	2.67 (1.27, 5.62)	<0.001	0.01
ln (Alb)	0.01 (0.00, 0.01)	0.01 (0.00, 0.05)	<0.001	<0.001
ln (Cr)	7.28 (3.06, 17.31)	7.23 (2.89, 18.10)	<0.001	<0.001
ln (CK)	3.29 (1.93, 5.61)	3.00 (1.60, 5.61)	<0.001	<0.001

*NLR, neutrophil-to-lymphocyte ratio; PLT, platelet; Alb, albumin; Tbil, total bilirubin; Cr, creatinine; CK, creatine kinase; ln, natural logarithm*.

### Predictive Nomogram for Severe COVID-19

**Figure 2:**
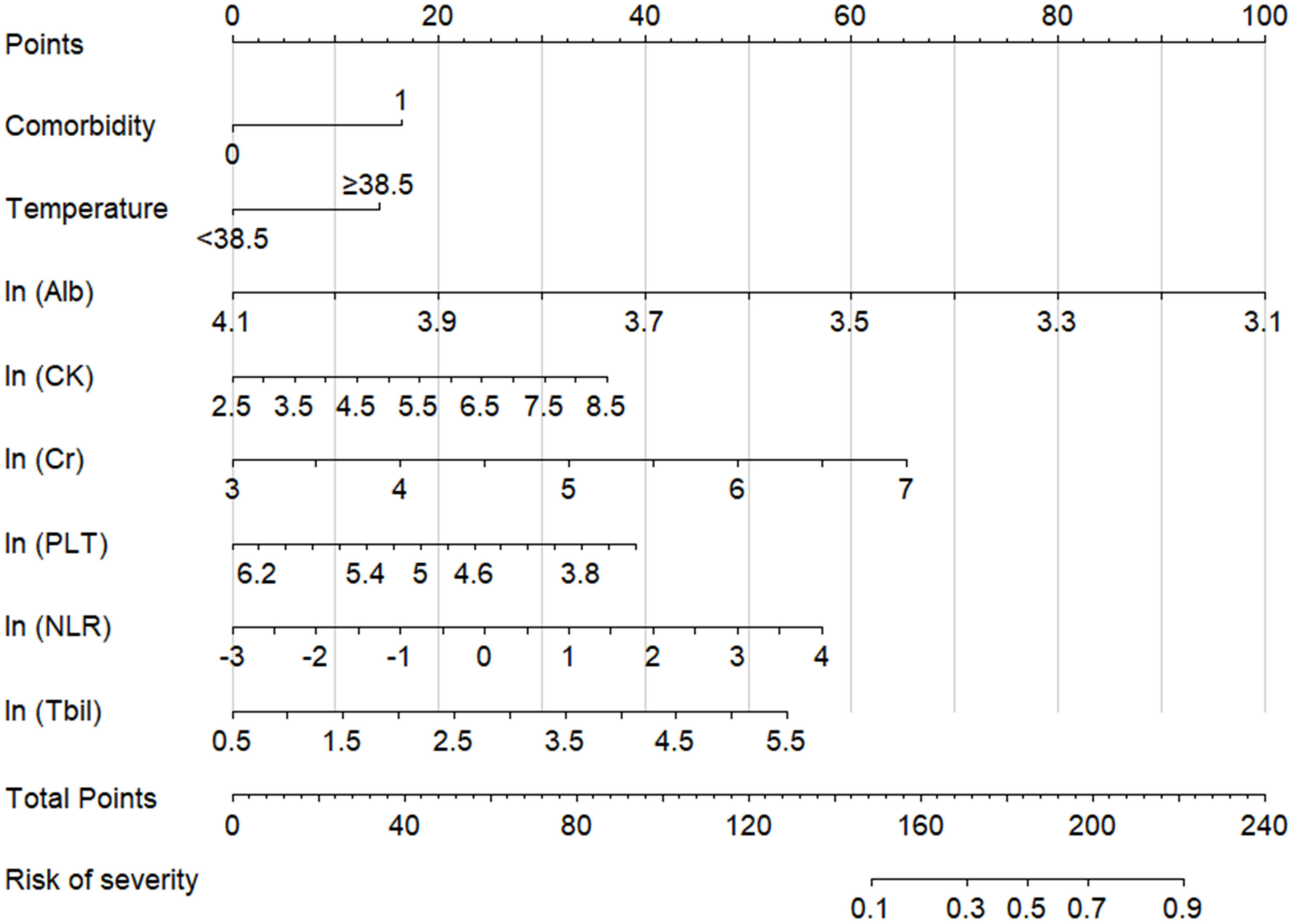
Nomogram model for assessing the risk of severe COVID-19. Total points are calculated by adding each point of the factors, such as comorbidities, temperature, ln (Alb), ln (CK), ln (Cr), ln (PLT), ln (NLR), and ln (Tbil), and the severity risk stratum of a patient can be obtained by projecting the total points downward. The higher that the score is, the higher that the probability of severe disease is. Regarding comorbidities, “0” indicates absent, and “1” indicates present. NLR, neutrophil-to-lymphocyte ratio; PLT, platelet; Alb, albumin; Tbil, total bilirubin; Cr, creatinine; CK, creatine kinase.

Based on the aforementioned eight variables, a nomogram model was established to predict the risk of severe COVID-19 (Figure 2) with a prediction accuracy of 85.9%, a leave-one-out cross validation accuracy of 81.6%, an AUROC of 0.858 (95% CI, 0.823–0.893), and a Hosmer-Lemeshow test *P*-value of 0.237.

### CANPT Score: A Novel Scoring Model for the Prediction of the Risk of Severe COVID-19

For convenience in clinical use, a novel scoring model was constructed based on the nomogram model and named the CANPT score, with scores ranging from 8 to 20 (Table 4). The AUROC of the CANPT score was 0.841 (95% CI, 0.804, 0.879), with a positive predictive value of 35.49% (95% CI, 30.23%, 41.13%), and a negative predictive value of 95.85% (95% CI, 92.81%, 97.68%) when 12 was used as the first cut-off value, and a positive predictive value of 69.64% (95% CI, 56.6%, 80.16%) and a negative predictive value of 85.36% (95% CI, 82.07%, 88.14%) when 16 was used as the second cut-off value (Table 5). In this study, 12/289 patients with a CANPT score <12 developed severe disease; 65/237 patients with a CANPT score ≥12 and <16 developed severe disease; and 39/56 patients with a CANPT score ≥16 developed severe disease. The actual incidence of severe disease in the COVID-19 patients with CANPT scores <12, ≥12 and <16, and ≥16 were 4.15, 27.43, and 69.64%, respectively. Thus, with cut-off values of 12 and 16, COVID-19 patients could be classified into low-risk, medium-risk, and high-risk groups with corresponding risks of developing severe COVID-19 of <5, 30, and 70%, respectively.

**Table 4:** Calculation of the CANPT and CAN scores.

**Variable**	**Adjusted OR (95% CI)**	***P*-value (Wald's test)**	**Points**
**CANPT score**			
Comorbidities			
Present	3.24 (1.98, 5.29)	<0.001	3
Absent			1
Highest temperature			
≥38.5°C	2.18 (1.30, 3.65)	0.003	2
<38.5°C			1
NLR			
≥3.7	1.90 (1.17, 3.11)	0.01	2
<3.7			1
PLT			
≥155			1
<155	1.95 (1.19, 3.18)	0.008	2
Alb			
≥38			1
<38	4.14 (2.49, 6.88)	<0.001	4
Tbil			
≥11	1.37 (0.82, 2.28)	0.229	2
<11			1
Cr			
≥85	3.13 (1.86, 5.29)	<0.001	3
<85			1
CK			
≥104	1.69 (1.03, 2.76)	0.036	2
<104			1
**CAN score**			
NLR			
≥7	4.92 (1.51, 16.10)	0.008	5
<7			1
Alb			
≥38			1
<38	2.50 (1.47, 4.24)	0.001	2
Cr			
≥80	8.62 (2.24, 33.24)	0.002	8
<80			1
CK			
≥106	4.34 (1.32, 14.28)	0.016	4
<106			1

*NLR, neutrophil-to-lymphocyte ratio; PLT, platelet; Alb, albumin; Tbil, total bilirubin; Cr, creatinine; CK, creatine kinase*.

**Table 5:** Accuracy of the CANPT score in estimating the risk of disease progression.

**Variable**	**Enrolled patients (*n* = 582)**
AUROC	0.841 (0.804, 0.879)
**Cut-off value (95% CI)**	12
Sensitivity, %	89.66 (82.65, 94.12)
Specificity, %	59.44 (54.92, 63.81)
Positive predictive value, %	35.49 (30.23, 41.13)
Negative predictive value, %	95.85 (92.81, 97.68)
Positive likelihood ratio	2.21 (2.12, 2.31)
Negative likelihood ratio	0.17 (0.16, 0.19)
**Cut-off value (95% CI)**	16
Sensitivity, %	33.62 (25.66, 42.64)
Specificity, %	96.35 (94.2, 97.75)
Positive predictive value, %	69.64 (56.6, 80.16)
Negative predictive value, %	85.36 (82.07, 88.14)
Positive likelihood ratio	9.22 (8.69, 9.78)
Negative likelihood ratio	0.689 (0.671, 0.706)

*AUROC, area under the receiver operating characteristic curve; CI, confidence interval*.

**Figure 3:**
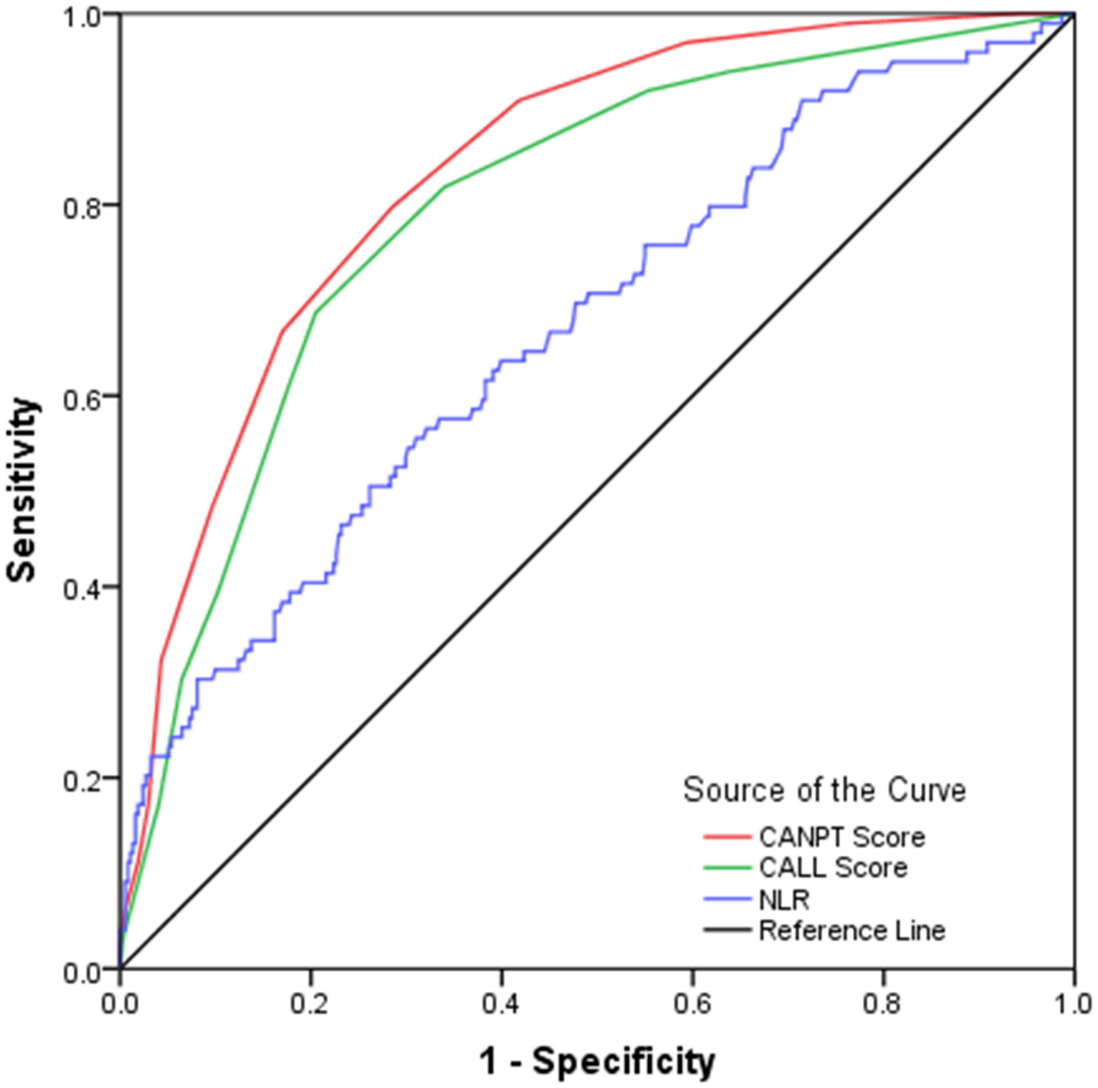
ROC curves of the CANPT score, CALL score, and the NLR. The area under the receiver operating characteristic curves of the CANPT score, CALL score, and the NLR were 0.835 (95% CI, 0.794, 0.876), 0.795 (95% CI, 0.747, 0.844), and 0.669 (95% CI, 0.607, 0.730), respectively. ROC, receiver operating characteristic curve; CI, confidence interval; NLR, neutrophil-to-lymphocyte ratio.

In this study, the CALL scores were calculated for 472 COVID-19 patients who had measurements of serum LDH levels on admission, and the predictive efficacies of the CALL score and the NLR were verified among these patients and compared with the efficacy of the CANPT score. The results showed that the AUROCs of the CANPT score, CALL score, and NLR were 0.835 (95% CI, 0.794, 0.876), 0.795 (95% CI, 0.747, 0.844), and 0.669 (95% CI, 0.607, 0.730), respectively. The predictive performance of the CANPT score was better than that of the CALL score and NLR (Figure 3).

### Model for the Prediction of the Risk of Mortality in COVID-19 Patients

Multivariate logistic regression analysis showed that the NLR and the levels of Alb, Cr, and CK were independent risk factors for mortality in patients with COVID-19 (Table 3). The CAN score was established to predict the risk of mortality in patients with COVID-19. The CAN score ranged from 4 to 19 (Table 4), with a prediction accuracy of 97.3%, a leave-one-out cross validation accuracy of 96.2%, an AUROC of 0.903 (95% CI, 0.832, 0.974), and a Hosmer-Lemeshow test *P*-value of 0.173. The cut-off value was determined by maximizing the Youden index at 15 points, with a sensitivity of 76.47% (95% CI, 52.23%, 90.95%), a specificity of 93.63% (95% CI, 91.28%, 95.38%), a positive predictive value of 26.53% (95% CI, 16.10%, 40.37%), a negative predictive value of 99.25% (95% CI, 98.01%, 99.78%), a positive likelihood ratio of 12.00 (95% CI, 11.12, 12.95), and a negative likelihood ratio of 0.25 (95% CI, 0.21, 0.29). In this study, 49/582 patients with COVID-19 had a CAN score ≥15; of these patients, 13 died. The actual mortality rates were 26.53% in the patients with a CAN score ≥15 and only 0.75% in those with a CAN score <15. Among the patients with severe disease, the actual mortality rate was 43.33% in the patients with a CAN score ≥15.

## Discussion

We enrolled 582 COVID-19 pneumonia patients in this study from four hospitals in three provinces, 74.57% of whom were from Hubei Province, and 25.43% of whom were from outside Hubei Province; thus, we reduced patient selection bias. Previous studies have shown that COVID-19 is a systemic disease with damage occurring not only in the lungs but also in many other systems, including the circulatory, cardiovascular, renal, gastrointestinal, endocrine, nervous, and integumentary systems (23, 24). In this study, some patients with COVID-19 had increased WBC and NE counts; increased ESRs; increased APTTs; increased levels of D-dimer, PCT, CRP, AST, GGT, BUN, Cr, CK, and LDH; decreased levels of HGB and Alb; and decreased PLT counts, further confirming the presence of multisystem damage in COVID-19 patients. Moreover, the incidence and degree of abnormalities in the above indicators in the patients with severe disease were significantly higher than those in the patients with non-severe disease, and the number of damaged systems and degree of damage were related to the severity of COVID-19. In this study, comorbidities, a body temperature ≥38.5°C, ln (NLR), ln (PLT), ln (Alb), ln (Tbil), ln (Cr), and ln (CK) were found to be independent risk factors for severe COVID-19, and the CANPT score comprehensively reflected the presence and degree of damage to the immune system, circulatory system, liver, kidneys, and heart in the patients with COVID-19, thereby accurately predicting the risk of severe disease.

Current studies have confirmed that patients with severe COVID-19 develop SARS-CoV-2-related cytokine storms and systemic inflammatory response syndrome (SIRS) (27–29). SIRS often leads to dysfunction in the lungs, kidneys, liver, heart, etc., and even multiple organ failure syndrome (MOFS). Angiotensin converting enzyme 2 (ACE2) and transmembrane protease serine 2 (TMPRSS2) are the receptors by which SARS-CoV-2 invades cells. In addition to the respiratory system, organs and tissues, such as the kidneys, heart, and bile duct epithelium, express ACE2/TMPRSS2 and therefore are potential target organs of SARS-CoV-2 that can be directly damaged (30, 31). Acute kidney injury (AKI) and myocardial injury have been observed in patients with critical COVID-19 (32–34). Therefore, organs, such as the kidneys, liver, and heart, can be affected by both direct damage from SARS-CoV-2 and indirect damage mediated by SIRS. Indicators of the function of these organs, such as Alb, Tbil, and Cr, were given relatively greater weight in the CANPT score to reflect that the degree of organ dysfunction plays an important role in the progression and severity of COVID-19.

Previous studies have shown that AKI is a common complication in patients with COVID-19 and that patients with kidney disease have a significantly higher risk of in-hospital mortality (35). Kidney biopsies from 17 patients with COVID-19 complicated with kidney injury did not show SARS-CoV-2 in the kidney tissue, suggesting that kidney injury in COVID-19 patients is mainly caused by SARS-CoV-2-associated SIRS, rather than direct renal damage caused by SARS-CoV-2 (36). A previous study found that the level of Cr is an independent risk factor for severe COVID-19; thus, Cr was given relatively greater weight in the CANPT score. Therefore, CANPT score could accurately reflect the extent of renal damage in COVID-19 patients early on admission.

Recent studies have shown that almost all hospitalized patients with COVID-19 have elevated levels of serum CK and LDH (28, 37, 38). Autopsies of patients who died of COVID-19 showed cardiomyocyte necrosis and monocyte infiltration (26). Persistently elevated CK indicates the occurrence and progression of myocardial injury in patients with COVID-19. CK is a prognostic marker for severe COVID-19, and the cut-off value is less than the upper limit of normal (ULN) (39). In this study, elevated CK levels were found in 14.14% of the patients with COVID-19 and was more common in patients with severe disease than those with non-severe disease (25.86 *vs*. 11.21%, *P* < 0.001). CK was given a weight of 2 in the CANPT score, reflecting myocardial damage in patients with COVID-19.

The activation of the coagulation system is very common in inflammatory and anti-inflammatory reactions and readily leads to diffuse intravascular coagulation (DIC), which plays an important role in the occurrence and development of organ damage (40). Abnormal coagulation function can be observed in patients with COVID-19, and almost all patients with severe COVID-19 have coagulation disorders. Several studies have shown that abnormal coagulation parameters are closely related to a poor prognosis of COVID-19 (41–46), especially D-dimer, which was not included in the modeling analysis due to the missing value of D-dimer being >10%. Previous studies have reported that the PLT count can be used as a marker of the progression of COVID-19 (47–51). In this study, a reduced PLT count was a risk factor for severe COVID-19, consistent with the results of previous studies. On the one hand, the reduced PLT count in patients with COVID-19 is due to the massive consumption of PLTs in the DIC process; autopsies of patients who died from COVID-19 showed that microthrombi formed in the pulmonary capillaries (26). On the other hand, SARS-CoV-2 can also directly infect bone marrow components, causing hematopoietic abnormalities or triggering an autoimmune response to blood cells (52, 53), further leading to a reduction in the PLT count. Therefore, the decrease in the PLT count reflects abnormal coagulation function, even DIC, in patients with COVID-19.

The presence of comorbidities is also an independent risk factor for severe COVID-19. Patients with hypertension, diabetes, structural lung disease, chronic kidney disease, etc., are more likely to develop ARDS and multiple organ dysfunction syndrome (MODS) in response to SIRS because of preexisting organ structural abnormalities and/or dysfunction (54–57). Due to the high levels of ACE2 expression on the surface, vascular endothelial cells may suffer from direct damage by SARS-CoV-2 and indirect damage due to SARS-CoV-2-associated SIRS. Patients with preexisting vascular endothelial cell damage due to diabetes, hypertension, or chronic kidney disease are predisposed to experiencing more damage to vascular endothelial cells by SARS-CoV-2, which could play a key role in the development of MOFS and severe disease (58).

In this study, a high NLR was a risk factor for severe disease in patients with COVID-19, consistent with the results of previous studies (10, 21, 59). The elevation in the NLR is related to immune disorders in patients with COVID-19. The invasion of SARS-CoV-2 triggers cytokine storms and SIRS in the body, resulting in an increased NE count. The LY count was found to be significantly reduced in peripheral blood from patients with COVID-19 due to recruitment and translocation in the local inflammatory system, which is especially pronounced in patients with severe disease (60). Another study found that SARS-CoV-2 could promote T lymphocyte apoptosis by activating the STAT1/IRF3 pathway (61). Thus, an elevated NE count and a reduced LY count in patients with COVID-19 lead to an increase in the NLR, reflecting the degree of the immune response and SIRS in patients with COVID-19.

Fever is a common symptom in patients with COVID-19. The immune response to SARS-CoV2 leads to systemic inflammation and even SIRS, with the consequent release of endogenous pyrogens, and the severity of fever represents the severity of SIRS (62). In this study, a body temperature ≥38.5°C was found to be more common in the group with severe disease than in the group with non-severe disease and was an independent risk factor for severe COVID-19.

ARDS is a clinical characteristic of severe COVID-19 and occurs in more than 71.2% of patients with severe COVID-19 in the ICU (63). The Murray score is used to evaluate the severity of acute lung injury and the risk of ARDS. The higher the Murray score, the more severe the acute lung injury, and the higher the risk of ARDS (64). Previous studies have shown that the Alb level in patients with COVID-19 is negatively correlated with the SARS-CoV-2 load and Murray score, and the higher the SARS-CoV-2 load in patients, the more critical the patient's condition (65). Recent studies have found that a low Alb level is an independent risk factor for disease progression in patients with COVID-19 (51, 66–68). In this study, a low Alb level was an important predictor of severe disease in patients with COVID-19 and was given a weight of 4 in the CANPT score, further validating previous research.

There have been reports of severe liver damage in patients with severe COVID-19 (69–71). Chai et al. suggested that liver injury in patients with COVID-19 could be caused by SARS-CoV-2-mediated injury in bile duct cells (72). However, SARS-CoV-2 has not been found in bile duct cells from patients who died of COVID-19, suggesting that liver damage in patients with COVID-19 is mediated by the SARS-CoV-2-associated cytokine storm and SIRS (73). Elevated ALT and AST levels suggest liver cell damage, while an elevated Tbil level indicates hepatocyte necrosis after the exclusion of bile duct obstruction or hemolysis. Several studies have found that an elevated Tbil level is significantly correlated with adverse outcomes of COVID-19 (69, 70, 74, 75); Tbil, which was given a weight of 2 in the CANPT score, reflects the severity of liver injury in patients with COVID-19.

Interestingly, the cut-off values of CK, Cr, and Tbil determined in this study were markedly lower than their ULNs. The rationale for CK, Cr, and Tbil levels being elevated is high risk for severe COVID-19, but the cut-off chosen for the CANPT score is lower than the ULN can be explained for the following reasons: (1) patients with higher values of CK, Cr, and Tbil than the cut-off levels could at high risk for according organ damage; and (2) higher values of CK, Cr, and Tbil may be resulted from an increase from much lower baseline levels due to organ injury; i.e., a significant increase in levels of CK, Cr, and Tbil could be important indicators of organ injury. The report from Qin et al. showed similar results in COVID-19 patients. In this study, cut-off values for high-sensitivity cardiac troponin I (hs-cTnI), creatine phosphokinase-MB (CK-MB), CK, and myoglobin (MYO) equivalent to ~49% ULN and a cut-off value of N-terminal pro-brain natriuretic peptide (NT-proBNP) equivalent of ~18.9% ULN were established for the prediction of adverse outcomes in COVID-19 patients, and patients with higher hs-cTnI, CK-MB, CK, MYO, and (NT-pro) BNP levels than the cut-off values were correlated with increased risk of death (39). Therefore, COVID-19 patients with CK, Cr, and Tbil levels higher than the cut-off values determined in this study, although within normal ranges, would still be at high risk for adverse outcomes and require more attention. Further, CANPT/CAN scoring could be helpful in identifying COVID-19 patients who are at high risk for severe disease.

In this study, <5% of the COVID-19 patients with a CANPT score <12 developed severe disease. Thus, the patients with a CANPT score <12 were considered low-risk patients, and the recommendation is to place these low-risk patients in a mobile cabin hospital or have them isolate at home with general symptomatic treatment with oral medication. In total, 27.43% of the patients with a CANPT score ≥12 and <16 developed severe disease and were considered at intermediate risk; therefore, people with a score within this range should be admitted to an isolation ward for respiratory monitoring and receive antiviral, anti-inflammatory, and symptomatic treatment. Nearly 70% of the patients with a CANPT score ≥16 developed severe disease. These patients should be considered at a high risk and should be transferred to an isolation ICU to receive comprehensive antiviral and symptomatic supportive treatment and respiratory support.

When the CAN score was used to predict the risk of mortality, only 0.75% of the patients with a CAN score <15 died, while among those with a CAN score ≥15, 26.53% of all patients, and 43.33% of the patients with severe disease eventually died. Thus, the CAN score could be used to identify patients who are at a high risk for mortality. Patients with a CAN score <15 are relatively safe, while those with a CAN score ≥15 are at a high risk for mortality regardless of whether they have severe disease and should be treated in the ICU.

The CALL score, which considers comorbidities, the LY count, age, and the LDH level, has been reported to have good predictive efficacy and is convenient for use in clinical practice (20). Studies have also reported that the NLR is an independent predictor of poor prognosis in patients with COVID-19 (21, 22). In this study, the CANPT score was compared with the CALL score and the NLR. The predictive performance of the CANPT score was significantly superior to that of the CALL score and the NLR with a larger AUROC.

The use of comorbidities and routine indicators, including body temperature, the NLR, the PLT count, and the levels of Alb, Tbil, Cr, and CK, renders the CANPT and CAN scores easy to calculate, and these scores are efficient in predicting the risk of severe illness and death in patients with COVID-19. These scores could be used to help clinicians to identify patients at high risk for poor outcomes or mortality soon after admission. Providing intensive care to the small proportions of patients who at high risk could improve their outcomes and reduce the mortality rate, and the rational allocation of limited medical staff and equipment could alleviate the serious shortages of medical resources. There were some limitations of this study. First, the practice for identification of severe and critically ill COVID-19 might differ in different hospitals, which could bias the results of the analysis. Second, this study only included Chinese patients; thus, the performance of the CANPT score and CAN score in patients of other ethnicities must be validated. Third, only 17 of the 582 COVID-19 patients included in this study died; therefore, the CAN model might not be sufficiently accurate to predict the risk of death in COVID-19 patients. Finally, this study was a retrospective study with a sample size of 582 without external validation, and the CANPT and CAN scores must be further validated in a large sample of patients from different regions.

The CANPT and CAN scores can be used to identify patients with COVID-19 who are at a high risk for severe disease or death soon after admission, guiding patient management and the rational allocation of limited medical resources based on patient risk stratification.

## Data Availability Statement

The data analyzed in this study is subject to the following licenses/restrictions: Clinical data were retrieved from the medical records databases of Shiyan Taihe Hospital, Ankang Central Hospital, Ningbo Hwamei Hospital, and Yichang Central People's Hospital. Requests to access these datasets should be directed to Yuanyuan Chen, cyy15871089714@163.com.

## Ethics Statement

The studies involving human participants were reviewed and approved by the Medical Ethics Committee of Shiyan Taihe Hospital. The approval number is 2020KS018. Written informed consent for participation was not required for this study in accordance with the national legislation and the institutional requirements.

## Author Contributions

YC and ZM designed and coordinated the research, contributed to the statistical analysis, and interpretation and the writing of the manuscript. ZM reviewed and edited the manuscript. JZ guided the data analysis. YC, HY, XZ, HH, ZJ, and SL collected the data. All authors contributed to and approved the submitted version of this manuscript.

## Conflict of Interest

The authors declare that the research was conducted in the absence of any commercial or financial relationships that could be construed as a potential conflict of interest.

## References

[1] Zhou P Yang XL Wang XG Hu B Zhang L Zhang W. A pneumonia outbreak associated with a new coronavirus of probable bat origin. Nature. (2020) 579:270–3. 10.1038/s41586-020-2012-7 32015507 PMC7095418

[2] Zhu N Zhang D Wang W Li X Yang B Song J. A novel coronavirus from patients with pneumonia in China, 2019. N Engl J Med. (2020) 382:727–33. 10.1056/NEJMoa2001017 31978945 PMC7092803

[3] Lu R Zhao X Li J Niu P Yang B Wu H. Genomic characterisation and epidemiology of 2019 novel coronavirus: implications for virus origins and receptor binding. Lancet. (2020) 395:565–74. 10.1016/S0140-6736(20)30251-8 32007145 PMC7159086

[4] Wu JT Leung K Leung GM. Nowcasting and forecasting the potential domestic and international spread of the 2019-nCoV outbreak originating in Wuhan, China: a modelling study. Lancet. (2020) 395:689–97. 10.1016/S0140-6736(20)30260-9 32014114 PMC7159271

[5] Li Q Guan X Wu P Wang X Zhou L Tong Y. Early transmission dynamics in Wuhan, China, of novel coronavirus-infected pneumonia. N Engl J Med. (2020) 382:1199–207. 10.1056/Nejmoa2001316 31995857 PMC7121484

[6] World Health Organization. Coronavirus disease 2019 (COVID-19) Situation report. Available online at: https://www.who.int/emergencies/diseases/novel-coronavirus-2019/situation-reports (accessed March 01, 2020).

[7] Zhang Y Chen Y Meng Z. Immunomodulation for severe COVID-19 pneumonia: the state of the art. Front Immunol. (2020) 11:577442. 10.3389/fimmu.2020.577442 33240265 PMC7680845

[8] Wynants L Van Calster B Collins GS Riley RD Heinze G Schuit E. Prediction models for diagnosis and prognosis of covid-19: systematic review and critical appraisal. BMJ. (2020) 369:m1328. 10.1136/bmj.m1328 32265220 PMC7222643

[9] Yuan M Yin W Tao Z Tan W Hu Y. Association of radiologic findings with mortality of patients infected with 2019 novel coronavirus in Wuhan, China. PLoS ONE. (2020) 15:e0230548. 10.1371/journal.pone.0230548 32191764 PMC7082074

[10] Cheng B Hu J Zuo X Chen J Li X Chen Y. Predictors of progression from moderate to severe coronavirus disease 2019: a retrospective cohort. Clin Microbiol Infect. (2020) 26:1400–5. 10.1016/j.cmi.2020.06.033 32622952 PMC7331556

[11] Ye W Chen G Li X Lan X Ji C Hou M. Dynamic changes of D-dimer and neutrophil-lymphocyte count ratio as prognostic biomarkers in COVID-19. Respir Res. (2020) 21:169. 10.1186/s12931-020-01428-7 32620118 PMC7332531

[12] Dong YM Sun J Li YX Chen Q Liu QQ Sun Z. Development and validation of a nomogram for assessing survival in patients with COVID-19 pneumonia. Clin Infect Dis. (2020) 10:ciaa963. 10.1093/cid/ciaa963 32649738 PMC7454485

[13] Yu C Lei Q Li W Wang X Liu W Fan X. Clinical characteristics, associated factors, and predicting COVID-19 mortality risk: a retrospective study in Wuhan, China. Am J Prev Med. (2020) 59:168–75. 10.1016/j.amepre.2020.05.002 32564974 PMC7250782

[14] Zheng Y Xiao A Yu X Zhao Y Lu Y Li X. Development and validation of a prognostic nomogram based on clinical and CT features for adverse outcome prediction in patients with COVID-19. Korean J Radiol. (2020) 21:1007. 10.3348/kjr.2020.0485 32677385 PMC7369204

[15] Cheng A Hu L Wang Y Huang L Zhao L Zhang C. Diagnostic performance of initial blood urea nitrogen combined with D-dimer levels for predicting in-hospital mortality in COVID-19 patients. Int J Antimicrob Agents. (2020) 56:106110. 10.1016/j.ijantimicag.2020.106110 32712332 PMC7377803

[16] Wu G Yang P Xie Y Woodruff HC Rao X Guiot J. Development of a clinical decision support system for severity risk prediction and triage of COVID-19 patients at hospital admission: an international multicenter study. Eur Respiratory J. (2020) 56:2001104. 10.1183/13993003.01104-2020 PMC7331655 32616597

[17] Wu Q Wang S Li L Wu Q Qian W Hu Y. Radiomics analysis of computed tomography helps predict poor prognostic outcome in COVID-19. Theranostics. (2020) 10:7231–44. 10.7150/thno.46428 32641989 PMC7330838

[18] Liang W Liang H Ou L Chen B Chen A Li C. Development and validation of a clinical risk score to predict the occurrence of critical illness in hospitalized patients with COVID-19. JAMA Intern Med. (2020) 180:1081–9. 10.1001/jamainternmed.2020.2033 32396163 PMC7218676

[19] Collins GS van Smeden M Riley RD. COVID-19 prediction models should adhere to methodological and reporting standards. Eur Respir J. (2020) 56:2020. 10.1183/13993003.02643-2020 32703773 PMC7377211

[20] Ji D Zhang D Xu J Chen Z Yang T Zhao P. Prediction for progression risk in patients with COVID-19 pneumonia: the CALL score. Clin Infect Dis. (2020) 71:1393–9. 10.1093/cid/ciaa414 32271369 PMC7184473

[21] Yang AP Liu JP Tao WQ Li HM. The diagnostic and predictive role of NLR, d-NLR and PLR in COVID-19 patients. Int Immunopharmacol. (2020) 84:106504. 10.1016/j.intimp.2020.106504 32304994 PMC7152924

[22] Yan X Li F Wang X Yan J Zhu F Tang S. Neutrophil to lymphocyte ratio as prognostic and predictive factor in patients with coronavirus disease 2019: a retrospective cross-sectional study. J Med Virol. (2020) 92:2573–81. 10.1002/jmv.26061 32458459 PMC7283791

[23] Gupta A Madhavan MV Sehgal K Nair N Mahajan S Sehrawat TS. Extrapulmonary manifestations of COVID-19. Nat Med. (2020) 26:1017–32. 10.1038/s41591-020-0968-3 32651579 PMC11972613

[24] Vabret N Britton GJ Gruber C Hegde S Kim J Kuksin M. Immunology of COVID-19: current state of the science. Immunity. (2020) 52:910–41. 10.1016/j.immuni.2020.05.002 32505227 PMC7200337

[25] Collins GS Wilkinson J. Statistical issues in the development of COVID-19 prediction models. J Med Virol. (2020) 93:624–5. 10.1002/jmv.26390 32749704 PMC7436733

[26] National Health Commission of the People's Republic of China. Guidance for 2019 Corona Virus Disease Prevention, Control, Diagnosis and Management. Available online at: http://books.ipmph.com/books/detail/2035540.shtml

[27] Wu D Yang XO. TH17 responses in cytokine storm of COVID-19: an emerging target of JAK2 inhibitor Fedratinib. J Microbiol Immunol Infect. (2020) 53:368–70. 10.1016/j.jmii.2020.03.005 32205092 PMC7156211

[28] Guzik TJ Mohiddin SA Dimarco A Patel V Savvatis K Marelli-Berg FM. COVID-19 and the cardiovascular system: implications for risk assessment, diagnosis, and treatment options. Cardiovasc Res. (2020) 116:1666–87. 10.1093/cvr/cvaa106 32352535 PMC7197627

[29] Tang Y Liu J Zhang D Xu Z Ji J Wen C. Cytokine storm in COVID-19: the current evidence and treatment strategies. Front Immunol. (2020) 11:1708. 10.3389/fimmu.2020.01708 32754163 PMC7365923

[30] Hoffmann M Kleine-Weber H Schroeder S Kruger N Herrler T Erichsen S. SARS-CoV-2 cell entry depends on ACE2 and TMPRSS2 and is blocked by a clinically proven protease inhibitor. Cell. (2020) 181:271–80 e278. 10.1016/j.cell.2020.02.052 32142651 PMC7102627

[31] Wan Y Shang J Graham R Baric RS Li F. Receptor recognition by the novel coronavirus from Wuhan: an analysis based on decade-long structural studies of SARS coronavirus. J Virol. (2020) 94:e00127–20. 10.1128/JVI.00127-20 31996437 PMC7081895

[32] Mehta P McAuley DF Brown M Sanchez E Tattersall RS Manson JJ. COVID-19: consider cytokine storm syndromes and immunosuppression. Lancet. (2020) 395:1033–4. 10.1016/S0140-6736(20)30628-0 32192578 PMC7270045

[33] Xu Z Shi L Wang Y Zhang J Huang L Zhang C. Pathological findings of COVID-19 associated with acute respiratory distress syndrome. Lancet Respiratory Med. (2020) 8:420–2. 10.1016/S2213-2600(20)30076-X 32085846 PMC7164771

[34] Tian S Xiong Y Liu H Niu L Guo J Liao M. Pathological study of the 2019 novel coronavirus disease (COVID-19) through postmortem core biopsies. Mod Pathol. (2020) 33:1007–14. 10.1038/s41379-020-0536-x 32291399 PMC7156231

[35] Cheng Y Luo R Wang K Zhang M Wang Z Dong L. Kidney disease is associated with in-hospital death of patients with COVID-19. Kidney Int. (2020) 97:829–38. 10.1016/j.kint.2020.03.005 32247631 PMC7110296

[36] Kudose S Batal I Santoriello D Xu K Barasch J Peleg Y. Kidney biopsy findings in patients with COVID-19. J Am Soc Nephrol. (2020) 31:1959–68. 10.1681/ASN.2020060802 32680910 PMC7461665

[37] Huang C Wang Y Li X Ren L Zhao J Hu Y. Clinical features of patients infected with 2019 novel coronavirus in Wuhan, China. Lancet. (2020) 395:497–506. 10.1016/S0140-6736(20)30183-5 31986264 PMC7159299

[38] Lippi G Lavie CJ Sanchis-Gomar F. Cardiac troponin I in patients with coronavirus disease 2019 (COVID-19): evidence from a meta-analysis. Prog Cardiovasc Dis. (2020) 63:390–1. 10.1016/j.pcad.2020.03.001 32169400 PMC7127395

[39] Qin JJ Cheng X Zhou F Lei F Akolkar G Cai J. Redefining cardiac biomarkers in predicting mortality of inpatients with COVID-19. Hypertension. (2020) 76:1104–12. 10.1161/HYPERTENSIONAHA.120.15528 32673499 PMC7375179

[40] Esmon CT. Possible involvement of cytokines in diffuse intravascular coagulation and thrombosis. Baillieres Best Pract Res Clin Haematol. (1999) 12:343–59. 10.1053/beha.1999.0029 10856974

[41] Dolhnikoff M Duarte-Neto AN de Almeida Monteiro RA da Silva LFF de Oliveira EP Saldiva PHN. Pathological evidence of pulmonary thrombotic phenomena in severe COVID-19. J Thromb Haemost. (2020) 18:1517–9. 10.1111/jth.14844 32294295 PMC7262093

[42] Lillicrap D. Disseminated intravascular coagulation in patients with 2019-nCoV pneumonia. J Thromb Haemost. (2020) 18:786–7. 10.1111/jth.14781 32212240 PMC7166410

[43] Tang N Li D Wang X Sun Z. Abnormal coagulation parameters are associated with poor prognosis in patients with novel coronavirus pneumonia. J Thromb Haemost. (2020) 18:844–7. 10.1111/jth.14768 32073213 PMC7166509

[44] Giannis D Ziogas IA Gianni P. Coagulation disorders in coronavirus infected patients: COVID-19, SARS-CoV-1, MERS-CoV and lessons from the past. J Clin Virol. (2020) 127:104362. 10.1016/j.jcv.2020.104362 32305883 PMC7195278

[45] Song JC Wang G Zhang W Zhang Y Li WQ Zhou Z. Chinese expert consensus on diagnosis and treatment of coagulation dysfunction in COVID-19. Mil Med Res. (2020) 7:19. 10.1186/s40779-020-00247-7 32307014 PMC7167301

[46] Ramlall V Thangaraj PM Meydan C Foox J Butler D Kim J. Immune complement and coagulation dysfunction in adverse outcomes of SARS-CoV-2 infection. Nat Med. (2020) 26:1609–15. 10.1038/s41591-020-1021-2 32747830 PMC7809634

[47] Henry BM de Oliveira MHS Benoit S Plebani M Lippi G. Hematologic, biochemical and immune biomarker abnormalities associated with severe illness and mortality in coronavirus disease 2019 (COVID-19): a meta-analysis. Clin Chem Lab Med. (2020) 58:1021–8. 10.1515/cclm-2020-0369 32286245

[48] Lippi G Plebani M Henry BM. Thrombocytopenia is associated with severe coronavirus disease 2019 (COVID-19) infections: a meta-analysis. Clin Chim Acta. (2020) 506:145–8. 10.1016/j.cca.2020.03.022 32178975 PMC7102663

[49] Bi X Su Z Yan H Du J Wang J Chen L. Prediction of severe illness due to COVID-19 based on an analysis of initial Fibrinogen to Albumin Ratio and Platelet count. Platelets. (2020) 31:674–9. 10.1080/09537104.2020.1760230 32367765 PMC7212543

[50] Bao C Tao X Cui W Yi B Pan T Young KH. SARS-CoV-2 induced thrombocytopenia as an important biomarker significantly correlated with abnormal coagulation function, increased intravascular blood clot risk and mortality in COVID-19 patients. Exp Hematol Oncol. (2020) 9:16. 10.1186/s40164-020-00172-4 32695551 PMC7366559

[51] Zhang K Liu X Shen J Li Z Sang Y Wu X. Clinically applicable AI system for accurate diagnosis, quantitative measurements, and prognosis of COVID-19 pneumonia using computed tomography. Cell. (2020) 181:1423–33 e1411. 10.1016/j.cell.2020.04.045 32416069 PMC7196900

[52] Yang M Ng MHL Li CK. Thrombocytopenia in patients with severe acute respiratory syndrome (review). Hematology. (2013) 10:101–5. 10.1080/10245330400026170 16019455

[53] Jolicoeur P Lamontagne L. Impairment of bone marrow pre-B and B cells in MHV3 chronically-infected mice. Adv Exp Med Biol. (1995) 380:193–5. 10.1007/978-1-4615-1899-0_33 8830480

[54] Chen Y Gong X Wang L Guo J. Effects of hypertension, diabetes and coronary heart disease on COVID-19 diseases severity: a systematic review and meta-analysis. medRxiv. 10.1101/2020.03.25.20043133

[55] Hespanhol V Bárbara C. Pneumonia mortality, comorbidities matter? Pulmonology. (2020) 26:123–9. 10.1016/j.pulmoe.2019.10.003 31787563

[56] Zou Q Zheng S Wang X Liu S Bao J Yu F. Influenza A-associated severe pneumonia in hospitalized patients: risk factors and NAI treatments. Int J Infect Dis. (2020) 92:208–13. 10.1016/j.ijid.2020.01.017 31978583

[57] Wang X Fang X Cai Z Wu X Gao X Min J. Comorbid chronic diseases and acute organ injuries are strongly correlated with disease severity and mortality among COVID-19 patients: a systemic review and meta-analysis. Research. (2020) 2020:2402961. 10.34133/2020/2402961 32377638 PMC7187729

[58] Varga Z Flammer AJ Steiger P Haberecker M Andermatt R Zinkernagel AS. Endothelial cell infection and endotheliitis in COVID-19. Lancet. (2020) 395:1417–8. 10.1016/S0140-6736(20)30937-5 32325026 PMC7172722

[59] Liu J Liu Y Xiang P Pu L Xiong H Li C. Neutrophil-to-lymphocyte ratio predicts severe illness patients with 2019 novel coronavirus in the early stage. J Transl Med. (2020) 18:206. 10.1186/s12967-020-02374-0 32434518 PMC7237880

[60] Diao B Wang C Tan Y Chen X Liu Y Ning L. Reduction and functional exhaustion of T cells in patients with coronavirus disease 2019 (COVID-19). Front Immunol. (2020) 11:827. 10.3389/fimmu.2020.00827 32425950 PMC7205903

[61] Zhu L Yang P Zhao Y Zhuang Z Wang Z Song R. Single-cell sequencing of peripheral mononuclear cells reveals distinct immune response landscapes of COVID-19 and influenza patients. Immunity. (2020) 53:685–96 e683. 10.1016/j.immuni.2020.07.009 32783921 PMC7368915

[62] Garami A Steiner AA Romanovsky AA. Fever and hypothermia in systemic inflammation. Handb Clin Neurol. (2018) 157:565–97. 10.1016/B978-0-444-64074-1.00034-3 30459026

[63] Yu Y Xu D Fu S Zhang J Yang X Xu L. Patients with COVID-19 in 19 ICUs in Wuhan, China: a cross-sectional study. Crit Care. (2020) 24:219. 10.1186/s13054-020-02939-x 32410714 PMC7223395

[64] Murray JF Matthay MA Luce JM Flick MR. An expanded definition of the adult respiratory distress syndrome. Am Rev Respir Dis. (1988) 138:720–3. 10.1164/ajrccm/138.3.720 3202424

[65] Liu Y Yang Y Zhang C Huang F Wang F Yuan J. Clinical and biochemical indexes from 2019-nCoV infected patients linked to viral loads and lung injury. Sci China Life Sci. (2020) 63:364–74. 10.1007/s11427-020-1643-8 32048163 PMC7088566

[66] Zhang J Wang X Jia X Li J Hu K Chen G. Risk factors for disease severity, unimprovement, and mortality in COVID-19 patients in Wuhan, China. Clin Microbiol Infect. (2020) 26:767–72. 10.1016/j.cmi.2020.04.012 32304745 PMC7159868

[67] de la Rica R Borges M Aranda M Del Castillo A Socias A Payeras A. Low albumin levels are associated with poorer outcomes in a case series of COVID-19 patients in Spain: a retrospective cohort study. Microorganisms. (2020) 8:94987. 10.1101/2020.05.07.20094987 32722020 PMC7463882

[68] Huang W Li C Wang Z Wang H Zhou N Jiang J. Decreased serum albumin level indicates poor prognosis of COVID-19 patients: hepatic injury analysis from 2,623 hospitalized cases. Sci China Life Sci. (2020) 63:1678–87. 10.1007/s11427-020-1733-4 32567003 PMC7306450

[69] Lei F Liu YM Zhou F Qin JJ Zhang P Zhu L. Longitudinal association between markers of liver injury and mortality in COVID-19 in China. Hepatology. (2020) 72:389–98. 10.1002/hep.31301 32359177 PMC7267515

[70] Phipps MM Barraza LH LaSota ED Sobieszczyk ME Pereira MR Zheng EX. Acute liver injury in COVID-19: prevalence and association with clinical outcomes in a large U.S. Cohort. Hepatology. (2020) 72:807–17. 10.1002/hep.31404 32473607 PMC7300739

[71] Parohan M Yaghoubi S Seraji A. Liver injury is associated with severe coronavirus disease 2019 (COVID-19) infection: a systematic review and meta-analysis of retrospective studies. Hepatol Res. (2020) 50:924–35. 10.1111/hepr.13510 32386449 PMC7273097

[72] Xu L Liu J Lu M Yang D Zheng X. Liver injury during highly pathogenic human coronavirus infections. Liver Int. (2020) 40:998–1004. 10.1111/liv.14435 32170806 PMC7228361

[73] Li J Fan JG. Characteristics and mechanism of liver injury in 2019 coronavirus disease. J Clin Transl Hepatol. (2020) 8:13–7. 10.14218/JCTH.2020.00019 32274341 PMC7132021

[74] Fu Y Zhu R Bai T Han P He Q Jing M. Clinical features of patients infected with coronavirus disease 2019 with elevated liver biochemistries: a multicenter, retrospective study. Hepatology. (2020). 10.1002/hep.31446 [Epub ahead of print]. 32602604 PMC7361581

[75] Chen LY Chu HK Bai T Tu SJ Wei Y Li ZL. Liver damage at admission is an independent prognostic factor for COVID-19. J Digest Dis. (2020) 21:512–8. 10.1111/1751-2980.12925 32713118

